# Endocannabinoids and Heterogeneity of Glial Cells in Brain Function

**DOI:** 10.3389/fnint.2016.00024

**Published:** 2016-07-05

**Authors:** Anja Scheller, Frank Kirchhoff

**Affiliations:** Molecular Physiology, Center for Integrative Physiology and Molecular Medicine, University of SaarlandHomburg, Germany

**Keywords:** astrocytes, microglia, endocannabinoid system, neuron-glia communication, glial heterogeneity

Attributable to their strong electrical activity, neurons have long been seen as the main determinants of brain function. Over the last decades, however, this view changed dramatically. A variety of specific roles have been assigned to different types of glial cells. Astrocytes constitute the link between the vascular system and neighboring neurons. They determine ion and transmitter homeostasis, metabolism and neuronal activity. Oligodendrocytes form the myelin sheath. They determine fast signal propagation, timing, and synchronicity. Microglial cells comprise not only the innate immune system of the brain, they also actively regulate synaptogenesis and removal of supra-numerous synapses. In general, microglial cells are quite uniformly distributed across different brain regions.

Looking at the system level of the brain, we have to take into account that the description of THE astrocyte as a uniform cell type is clearly outdated. Exploring astrocyte heterogeneity based on localization, function, age, and condition is becoming a major endeavor to fully understand brain function (Oberheim et al., 2012; Bayraktar et al., 2015; Schitine et al., 2015; Bribián et al., 2016). Astrocyte heterogeneity is not only a phenomenon between different brain regions such as cortex, hippocampus, or cerebellum, but also within a given territory. In the healthy, adult cortex the astroglial intermediate filament protein GFAP (glial fibrillary acidic protein) can be hardly detected in most of the astrocytes and only those contacting brain vasculature express significant levels (Figure 1A). In contrast, in the hippocampus almost all astrocytes exhibit a strong and steady expression (Figure 1C). Another striking example of astroglial diversity is reflected by the expression of various transporters or transmitter receptors. Perisynaptic appendages of cerebellar Bergmann glia are morphologically hard to distinguish from hippocampal astrocyte processes at the ultrastructural level. But, while the first glial cell type is characterized by high levels of AMPA-type glutamate receptor expression, the latter is completely devoid of these receptors (Matthias et al., 2003; Saab et al., 2012). Similar to the heterogeneity of astrocytes within or between given brain regions, we also have to consider a heterogeneity within a single cell given by the highly complex and polarized morphology of astrocytes bridging the gap from the brain capillaries to the neuronal synapses.

**Figure 1:**
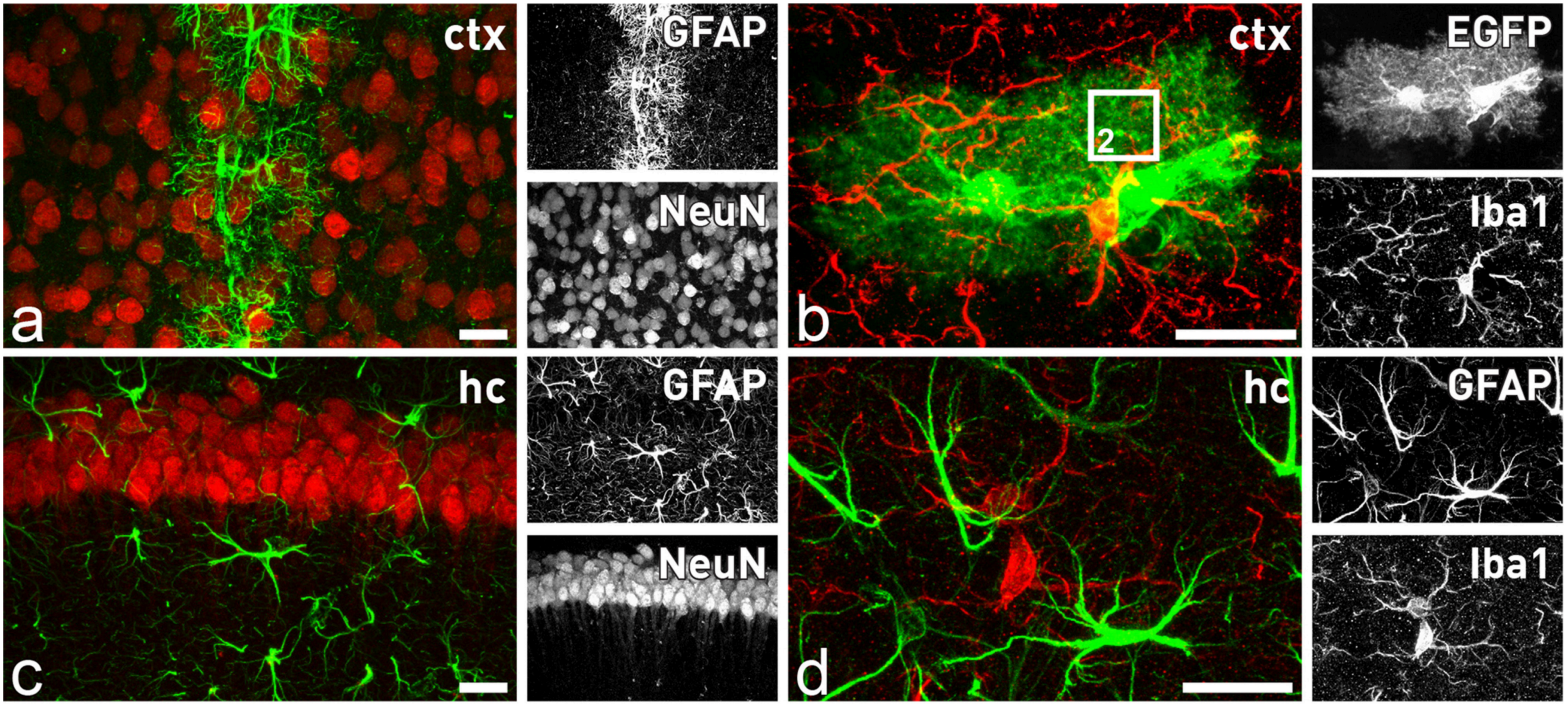
**Astrocytes and microglia in the forebrain**. Distinct subtypes of astrocytes are present throughout the brain, while microglial cells seem to be more homogenously distributed. In the cortex only astrocytes in close contact to blood capillaries express significant levels of GFAP **(A)**, while all astrocytes are closely intermingled with adjacent neurons **(A)** or microglia **(B)**. In contrast, in the hippocampus all astrocytes express GFAP **(C)**. They are also in close contact to neurons **(B)** and microglial cells **(D)**. Comparison of GFAP staining **(D)** with EGFP expression in **(B)** of TgN (GFAP-EGFP)_GFEC_ transgenic mice reveals only in the latter the fine arborization of perisynaptic and perivascular astrocytic processes. The square in **(B)** indicates the magnified view that is schematically depicted in Figure 2. Scale bars indicate 20 μm.

Taking into account that a cortical astrocyte contacts up to 600 dendrites, the broad and extended impact of astrocytes on neuronal plasticity becomes evident (Heller and Rusakov, 2015). It is not too tempting to speculate that this feature of astrocytes is less involved in the integration of neuronal signals rather than in modulation and synchronization of neuronal network activity of adjacent microcircuit domains of defined central nervous system (CNS) regions. While astrocytes can directly affect local synapses in the close neighborhood (<20 μm), the gap junction-coupled astroglial syncytium can bridge neighboring microcircuits (Figure 2; Navarrete and Araque, 2010; Navarrete et al., 2014).

**Figure 2:**
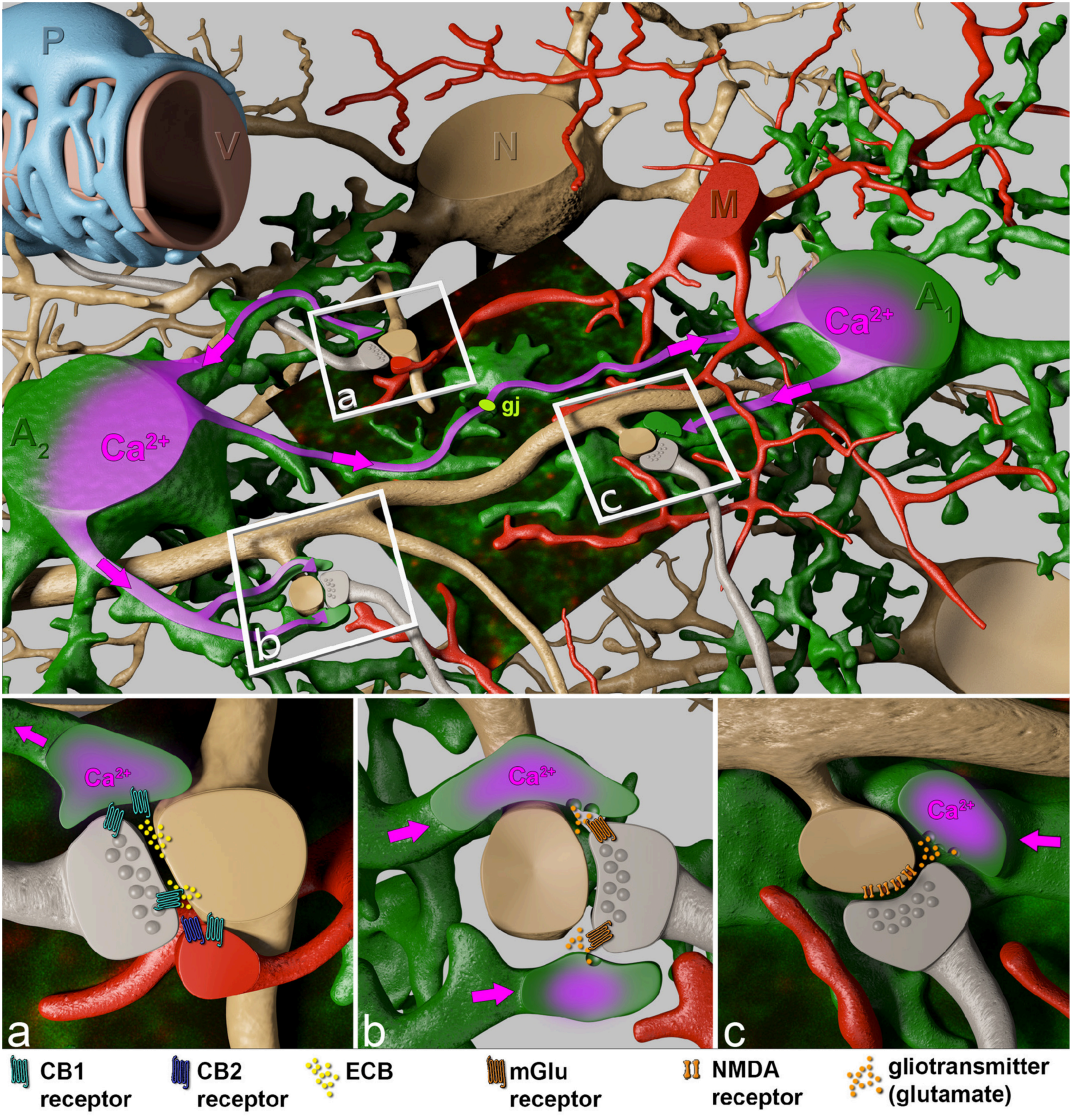
**Close interactions of perisynaptic astroglial and microglial processes with the synaptic elements of adjacent neurons**. The boxed regions indicate specific sites of neuron-glial interactions. Perisynaptic astroglial processes (green) express the cannabinoid receptors CB1 (cyan), while microglial processes both, CB1 (cyan) and CB2 (blue) receptors in close contact to postsynaptic ECB (yellow) release sites **(A)**. Astrocytes respond to ECB via CB1 receptors with an increase of intracellular Ca^2+^ (purple). This intracellular Ca^2+^ rise spreads (pink arrows) through the astrocyte towards distant synapses **(B)**, where Ca^2+^-evoked release of the gliotransmitter glutamate (orange) affects neuronal physiology via presynaptic metabotropic glutamate receptors (mGluR1, brown), and generates a persistent synaptic change **(B)**. In addition, the Ca^2+^ wave can be propagated through the gap junction-coupled astroglial syncytium (gj, light green) where on even more distant perisynaptic processes gliotransmitters (glutamate) are released as response to postsynaptic ECB liberation. Subsequently, gliotransmitters can act on postsynaptic NMDA receptors (**C**, brown) inducing slow inward currents.

Common to all glial cells is the expression of a similar set of ionotropic or metabotropic receptors as their adjacent neuronal counterparts. And indeed, glutamate, GABA and ATP have been studied intensively, not only as classical transmitters but also as important molecular entities that constitute various forms of bidirectional communication among neurons and glia. Quite surprisingly, however, the most abundant metabotropic G-protein coupled receptor of the brain is sensitive to none of these important molecules. It is the cannabinoid type I (CB1) receptor which is expressed at variable levels on almost all cells of the CNS and is activated endogenously by two metabolites of arachidonic acid, N-arachidonoyl-ethanolamine (anandamide, AEA) and the more potent 2-arachidonoyl-glycerol (2-AG; Stella, 2010; Boorman et al., 2016). More commonly known is their relative which is found in *Cannabis sativa*, Δ9-tetrahydrocannabinol (THC), the main constituent of marijuana. Like THC, also AEA and 2-AG are psychoactive. While the CB1 receptor is expressed quite uniformly, the cannabinoid type II (CB2) receptor is expressed at low levels, but strongly activated in microglia or endothelial cells in CNS pathologies (Herkenham et al., 1990; Piomelli, 2003; Núñez et al., 2004; Atwood and Mackie, 2010; Zhang et al., 2014; Boorman et al., 2016; Oliveira da Cruz et al., 2016). The lipophilic nature of the endocannabinoids (ECB) together with the broad expression of the CB1 receptor results in more generalized functions in all brain regions. Dependent on the region-specific pattern of neurons and glial cells, activation of the endogenous cannabinoid signaling system can affect numerous neural circuits broadly, ranging from cognition to eating or motor behavior. Here, we would like to discuss the specific functions of CB1 and CB2 receptors on the two glial cell types, astrocytes and microglia in respect to the more recently described cellular heterogeneity.

Frequent use of marijuana by distinct human populations had provided strong insight into the function of the ECB system, the receptors as well as their ligands. Cannabis users exhibited significant distortions of their working and declarative memory. The impaired reality monitoring further resulted in a distinct susceptibility to false memories (Riba et al., 2015). In more controlled animal experiments using rodents, THC induced a combination of physiological/behavioral changes including spontaneous activity, catalepsy, hypothermia, and analgesia (Little et al., 1988; Howlett et al., 2002). Due to distinct expression of the respective receptors, CB1 and CB2, ECB signaling can determine brain functions at different levels. While expression of the CB1 receptor is held responsible for the more psychoactive behavior after activation, the CB2 receptor is more involved in anti-inflammatory processes (Buckley et al., 2000; Mackie, 2005; Buckley, 2008).

In contrast to excitatory transmitters, ECBs are generated and released from activated post-synaptic dendritic terminals and evoke a diversity of complex signaling routes involving neurons and adjacent glia (see Figure 2): (1) They act retrogradely at neuronal pre-synapses to control further transmitter release, resulting in *suppression* of excitation (Navarrete et al., 2014). (2) Simultaneously activated CB1 receptors on perisynaptic astroglial processes, however, cause an intracellular Ca^2+^ release from internal stores via the G_q∕11_ / phospholipase C / inositol trisphosphate pathway (Navarrete and Araque, 2008, 2010) and stimulate additional release of gliotransmitters, preferentially glutamate, *triggering* presynaptically localized metabotropic glutamate receptors (Figure 2B) as well as postsynaptic NMDA receptors (Figure 2C). The depression of excitatory neurotransmission by ECB-evoked presynaptic inhibition of neurotransmitter release impairs spatial working memory (Misner and Sullivan, 1999; Carlson et al., 2002; Takahashi and Castillo, 2006; Bajo et al., 2009; Schoeler and Bhattacharyya, 2013; Schoeler et al., 2016). This inhibiting signaling only works over short distances of less than 20 μm (Navarrete and Araque, 2010). In contrast, ECB-evoked signals in adjacent astroglial processes can affect remote synapses by using the gap junction-coupled astroglial syncytium as a bridge (Navarrete and Araque, 2010; Navarrete et al., 2014; Gómez-Gonzalo et al., 2015; Figure 2). Interestingly, the CB1-mediated astroglial release of glutamate can cause both, potentiation as well as depression of neuronal transmission. In the hippocampus, activation of postsynaptic NMDA receptors (Figure 2C) induces slow inward currents in CA1 pyramidal neurons (Navarrete and Araque, 2008; Navarrete et al., 2013, 2014), while presynaptic NMDA receptor activation causes spike timing-dependent depression (Min and Nevian, 2012). Presynaptic activation of type 1 metabotropic glutamate receptors (mGluR1) coincident with NO signaling from the postsynapse induces long-lasting synaptic potentiation. mGluR-mediated activation of presynaptic protein kinase C enhances transmitter release persistently (Gómez-Gonzalo et al., 2015). The ECB signaling on astrocytes induces highly selective, circuit-specific modulation of synaptic transmission. In the striatum the astroglial glutamate release acts only on the same subtype of medium spiny neuron (MSN) from which the ECB was released (Martin et al., 2015). The neuronal subtypes can be distinguished by their dopamine receptor expression (D1 and D2). The ECB releasing MSN and the glia-modified neuron have both to express either the D1 or the D2 receptor; no potentiation is detected if one MSN expresses the D1 and the other the D2 receptor or vice versa.

In the hippocampus, the maintenance of epileptic discharges is reduced when the neuron-to-astrocyte communication via CB1 receptor activation is pharmacologically blocked (Coiret et al., 2012). Surprisingly, despite the fact that the CB1 receptor is widely expressed on all hippocampal cells, it was only the astrocyte specific deletion of the CB1 receptor gene that completely eliminated THC-induced depression (Han et al., 2012). In detail, THC stimulates glutamate release from astrocytes by activation of its CB1. The adjacent neuron then shows long-term depression (LTD) by internalizing its AMPA-type glutamate receptors. At the behavioral level, a severe impairment of spatial working memory is observed (Han et al., 2012). But CB1 receptor expression in astrocytes is not restricted to processes at synapses. Astrocytes are also in close contact to blood vessels where the CB1 receptor has been localized to the perivascular endfeet as well (Rodriguez et al., 2001). The functional meaning for this spatial separation is not yet clear. Obviously, the function of the astroglial CB1 receptor is not restricted to neuronal transmission. By controlling local cerebral blood flow, astrocytes adjust the energy supply within a single neuronal microcircuit or even linking adjacent networks, a phenomenon that has been termed neurovascular coupling (Stella, 2010). The modulation of neurovascular coupling by targeting CB1 receptors could become important in novel strategies to combat the sequelae of ischemic insults. Similarly, it will be highly interesting to assign distinct roles of CB1 receptors which are expressed on perisynaptic processes or at the perivascular endfeet to specific behaviors. So far, only learning paradigms have been tested which would favor more the influence of CB1 receptors at the synapse, e.g., the spatial working memory in the hippocampus investigated by Han et al. (2012). It would now be very interesting, though technically challenging, to perform two-photon imaging of the neurovascular unit in experimental mice under different conditions of genetic or pharmacological CB1 receptor modulation and cognitive stress. Curiously, these experiments could be done by the same genetically modified mice (GFAP-CreERT2 × floxed CB1) that Han et al. (2012) had investigated. The GFAP-CreERT2 mouse line shows a more efficient recombination of cortical astrocytes that are part of the neurovascular unit and contact the capillaries (Jahn et al., 2015).

Another important glial cell type involved in ECB signaling are microglia. Their processes that are also in close contact with synapses and blood vessels express both, CB1 and CB2 receptors (Núñez et al., 2004; Maresz et al., 2005; Cabral et al., 2008; Figures 1B,D). While these innate immune cells of the CNS express only very low levels of the CB1 receptor, their major player of the ECB signaling game is the CB2 receptor. Under resting conditions the CB2 receptor is weakly expressed as well, but expression levels are highly responsive and get strongly increased upon neuroinflammatory processes associated with brain pathologies (Maresz et al., 2005; Cabral et al., 2008; Atwood and Mackie, 2010; Mecha et al., 2015; Schmole et al., 2015). Interestingly, in contrast to the CB1 receptor, selective agonists of the CB2 receptor are not psycho-active. Instead, the most potent ECB, 2-AG, exhibits strong neuroprotective effects in acute CNS injuries (Ashton and Glass, 2007; Arevalo-Martin et al., 2010). Triggering the microglial CB2 receptor reduces the release of pro-inflammatory cytokines by activated microglia. And similar to astrocytes, there is also a distinct population of microglia that surround the brain capillaries. The perivascular microglia closely interact with the capillary-forming endothelial cells that express CB2 receptors as well. And indeed, pharmacologically selective stimulation of the CB2 receptor stabilized and enhanced the efficacy of the blood-brain barrier (BBB), thereby dampening the consequences of neuroinflammatory injuries (Ramirez et al., 2012). In addition, activation of CB2 receptors signaled into the luminal side of the endothelium and reduced the homing of leukocytes to even further rescue an inflammatory response by recruiting peripheral immune cells, as it could be visualized by repeated long-term two-photon microscopy (Ramirez et al., 2012).

## Outlook

Obviously ECB signaling in the brain comes in different glial flavors. While CB1 receptors of perisynaptic astroglial process strongly affect different forms of neuronal plasticity, microglial and endothelial CB2 receptors provide efficient neuroprotection by reducing neuroinflammatory processes including tightening of the BBB. However, important research questions remain for the future:

What is the function of astroglial CB1 receptors at the perivascular endfeet? In this context it is particularly intriguing that CB1 receptors are not only widely expressed throughout the brain on the cell surface, but also on mitochondrial membranes. Could it be that ECB signaling represents a major regulatory system that regulates energy demands in the brain, acting on a variety of different levels from regulating glucose uptake at the brain vasculature to fine-tuning oxidative phosphorylation in mitochondria?

Does the low level of the CB2 receptor on microglia contribute to normal brain functions? Are there synergistic interactions of the individual components of ECB signaling on different cell types? More cell-specific genetic manipulations of ECB signaling are required. In particular, specific receptor targeting as well as imaging approaches, that will help to unravel the diversity of intracellular signaling cascades, are necessary. Innovative combination of imaging and genetic approaches *in vivo* will pave the way for exciting new findings.

## Materials and methods

This study was carried out at the University of Saarland (Center for Integrative Physiology and Molecular Medicine, CIPMM) in strict accordance with recommendations of European and German guidelines for the welfare of experimental animals. Animal experiments were approved by Saarland state's “Landesamt für Gesundheit und Verbraucherschutz” in Saarbrücken/Germany (animal license number: 71/2010). No vulnerable populations (minors, persons with disabilities or endangered animal species) were involved.

Mouse breeding and animal experiments were performed at the animal facility and the research labs of the CIPMM. For the immunohistochemical analysis heterozygous 8-week-old TgN(hGFAP-EGFP)_GFEC_ mice were used (Hirrlinger et al., 2005). Mouse perfusion, tissue fixation and vibratome slice preparation (40 μm) were performed as described previously (Huang et al., 2014). For immunohistochemistry, the following antibodies were used: polyclonal rabbit anti-GFAP (1:1000, Dako Cytomation, Glostrup, Denmark) and anti-Iba1 (1:1000, Wako, Richmond, USA), monoclonal mouse anti-NeuN (1:500, Merck Millipore, Darmstadt, Germany) and anti-rabbit/mouse antibody conjugated Alexa543/633 (1:2000, Invitrogen, Grand Island NY, USA). The transgenic EGFP signal was directly recorded without additional antibody enhancement. Confocal images were taken by a laser-scanning microscope (LSM-710, Zeiss), processed with ZEN software (Zeiss) and displayed as maximum intensity projections. Figures presented in this work were modified with image processing tools of ImageJ (Fiji, www.fiji.sc).

## Author contributions

All authors listed, have made substantial, direct and intellectual contribution to the work, and approved it for publication.

## Funding

Research of the authors is supported by grants from the Deutsche Forschungsgemeinschaft DFG (SFB 894, SPP 1757, FOR 2289), the European Union (ERA-NET Neuron BrIE), the ARSEP foundation and the HOMFOR programme of the University of Saarland Medical Faculty.

### Conflict of interest statement

The authors declare that the research was conducted in the absence of any commercial or financial relationships that could be construed as a potential conflict of interest.
